# Did We Get Our Money’s Worth? Bridging Economic and Behavioral Measures of Program Success in Adolescent Drug Prevention

**DOI:** 10.3390/ijerph10115908

**Published:** 2013-11-08

**Authors:** Kevin N. Griffith, Lawrence M. Scheier

**Affiliations:** 1 Research Facilitation Team (RFT), Army Analytics Group, 20 Ryan Ranch Road, Suite 290, Monterey, CA 93940, USA; E-Mail: kevin.griffith8@gmail.com; 2 Positive Psychology Center/RFT, University of Pennsylvania, Philadelphia, PA 19104, USA

**Keywords:** opportunity cost, cost benefit, valuation, cost effectiveness, program efficacy, statistical mediation, economic analysis

## Abstract

The recent U.S. Congressional mandate for creating drug-free learning environments in elementary and secondary schools stipulates that education reform rely on accountability, parental and community involvement, local decision making, and use of evidence-based drug prevention programs. By necessity, this charge has been paralleled by increased interest in demonstrating that drug prevention programs net tangible benefits to society. One pressing concern is precisely how to integrate traditional scientific methods of program evaluation with economic measures of “cost efficiency”. The languages and methods of each respective discipline don’t necessarily converge on how to establish the true benefits of drug prevention. This article serves as a primer for conducting economic analyses of school-based drug prevention programs. The article provides the reader with a foundation in the relevant principles, methodologies, and benefits related to conducting economic analysis. Discussion revolves around how economists value the potential costs and benefits, both financial and personal, from implementing school-based drug prevention programs targeting youth. Application of heterogeneous costing methods coupled with widely divergent program evaluation findings influences the feasibility of these techniques and may hinder utilization of these practices. Determination of cost-efficiency should undoubtedly become one of several markers of program success and contribute to the ongoing debate over health policy.

## 1. Introduction—School-Based Drug Abuse Prevention

Backed by substantial government funding and a national public health agenda, the past three decades have witnessed a proliferation of school-based drug prevention programs. The combined financial outlay for individual states and the federal government totaled nearly US$2 billion on substance abuse prevention in 2005 [1]. Impetus for schools to adopt drug prevention rests in part with the 1994 Elementary and Secondary Education Act (Title IV, Sections 41, 114, 116, 20 U.S.C. 71, 117, 116), more commonly known as the Safe and Drug Free Schools and Community Act, as well as growing societal concerns over the problem of youth drug use. Core components of the Act facilitate implementation of drug prevention, collaboration among community, school, and health service providers to ensure that students can achieve their academic goals in a safe and drug-free environment. The Act also stipulates that schools use evidence-based programs and sets forth stringent research design, psychometric, and statistical criteria to ensure adequate state-of-the-art program evaluation. The emphasis on using evidence-based programs based on stringent evaluation criteria has promoted the search for “*what works*” and “*for whom*” [2,3,4]. For prevention researchers the question of efficacy primarily involves determining whether the program works based on accepted statistical conventions and evaluation principles [5]. This entails manipulation checks that determine whether programs work in the manner hypothesized [6,7]. Standard evaluation criteria include assessing whether intervention strategies mollify risk (or boost protection), which in turn influences behavioral outcomes.

In the field of prevention science, tests of statistical mediation center on ascertaining the “*active ingredients*” of behavior change [8]. A social skills training program, for instance, would test whether youth demonstrate more assertiveness, communication, and drug refusal skills following exposure to a cognitive-behavioral intervention, and importantly, whether improved skills is protective against drug initiation [9,10]. Such tests follow published standards for testing statistical mediation [11,12], which ascertain the magnitude of the mediated effect. These tests have undergone considerable scientific scrutiny over the years and have been refined to include the Sobel multivariate delta method [13]. This latter approach provides an “inferential” coefficient (with confidence intervals) of the main product terms deemed essential to establish credible evidence of mediation. Indeed, programs that incorporate traditional “*mediation*” designs are now required fare for prevention science and guided by newly published and widely disseminated guidelines [5].

Even with statistical mediation required as a *condicio sine qua non* for evidence-based prevention, there are still many pitfalls associated with using blanket statistical approaches to determine what works. For one thing, consistent with the operational definitions and different tiers of prevention, universal programs attempt to favorably influence a large body of students; providing them with appropriate preventive tools (*i.e.*, resistance skills) before the conditions of risk have their effect [14,15]. Treatment exposure may favorably affect some but not all youth given individual differences in pre-existing conditions of risk (and or protection) and/or exposure to the behaviors in question (e.g., drug use or negative peer influences). As a result, program effectiveness may be seriously reduced or overstated with conventional mediation analyses that apply a blanket “one-size fits all” program evaluation. As an illustration, Ellickson and colleagues showed the effect of prior risk calibrates program effectiveness on drug use with a large school-based prevention program [16]. Botvin and colleagues showed that program effects were best framed by a measure of “dose” or implementation fidelity with students receiving over 60% of the intervention faring best [9]. In these instances, more granular mixture analyses that define clusters or “classes” of youth who differentially respond to treatment may be required [17]. Even with these caveats, the best way to show whether a program works is to test the theoretical and developmental postulates guiding formulation of the intervention model. These tests involve statistical mediation among several other alternative approaches to ensure program effects affecting developmental risk processes lead to behavior change.

Following this lead, numerous program evaluations have provided empirical confirmation of statistical mediation for school-based drug programs [18,19,20,21,22]. Many of these exemplary studies used randomized control trials to reinforce program effects on target risk mechanisms and designated outcomes including tobacco, alcohol, and marijuana use. The combination of randomized trials, considered the gold standard of experimental methods [23], with mediation provides a rigorous means to address the action theory underlying drug prevention [12,24]. Unfortunately, other factors also predicate whether a program “*works*” and from a policy perspective, nets the desired outcomes. In traditional economic terms, this involves determining whether a program is “*cost-effective*”. In other words, merely because a program works does not mean it will be widely adopted or used given the program can be costly, difficult to implement, and require incredible personnel, logistic, and financial resources. In keeping with economic theories of consumption, budget decisions may factor in heavily whether a school district purchases a program regardless of the program developer’s marketing savvy. As several national surveys have shown, this can lead to the adoption of cheaper, if not equally effective programs [25].

In order to address these pressing concerns, this paper examines two interlocking components of school-based, drug prevention program evaluation. The first component elucidates economic strategies for program evaluation, providing the reader with a basic understanding of how to conduct economic analyses of school-based prevention programs. This section includes clarification of terminology, the rationale, methodology, and standard practices for conducting economic analysis, derivation of cost and benefit estimates, and a description of non-traditional factors that influence economic analysis. The material covered in this section also provides relevant examples of program evaluation for school-based drug prevention using economic techniques. Although a great deal of program evaluation and economic information has been accumulated regarding alternative “family-based” programs, the focus of this paper rests with programs delivered in school settings, using either entire schools or classrooms as the unit of assignment with corresponding unit of observation at the student level. The paper concludes with a discussion of blending traditional behavioral program evaluation with economic analyses. This latter discussion seeks to produce a synthesis that has ramifications for social and public policy and the application of school-based drug prevention nationwide. 

## 2. Program Evaluation from an Economic Perspective

To begin this discussion, opportunity cost is one of the fundamental concepts in economic analysis [26,27]. Opportunity cost represents the potential benefits to society that could have been realized if funding had been used for other potential alternatives. From an economic point of view, opportunity costs are uniquely different from accounting costs, which encompass monetary expenditures restricted to provision of services and direct cost expenditures (e.g., salary for teachers and capital costs). Prevention programs create opportunity costs because they consume scarce resources that could have been used for other societal purposes—that is, money that could instead have been spent on improving education, repairing infrastructure, conducting medical research, and other desired quality of life improvements. In making the decision whether or not to fund these programs, policymakers are essentially deciding whether their constituents will relinquish the potential advantages that derive from one program for another [28].

In deciding whether one program will net better outcomes than another, skeptics have raised concerns about whether prevention is efficacious or cost effective [28,29,30]. Researchers and program administrators are increasingly being asked to justify expenditures on competing substance abuse prevention and treatment programs, and economic analysis is one of the primary tools at their disposal to demonstrate that their respective programs are worthy of public investment and consumption [5,31].

### 2.1. The Case for Economic Analysis

Despite the increasing press to justify public expenditures, prevention programs are rarely subjected to a rigorous economic analysis [32]. This trend is not unique to drug prevention, but is endemic to clinical research and program evaluation in general [33]. There are several factors that may dampen enthusiasm for implementing economic analysis. First, researchers may intrinsically believe that their programs are cost-effective, precluding further analysis [28,34,35]. This mindset revolves around the standpoint suggesting that “irrespective of cost, if the program protects youth from harm then it benefits society.” Second, some school-based programs do not collect adequate data to facilitate economic analysis. For example, the ALPHA drug prevention program, a selective multi-modal intervention targeting “at-risk” elementary school children (children were selected for the one-semester program based on prescreen cut-off scores for low self-esteem, learning deficits, and conduct problems) did not separate program costs from school-related costs leaving researchers to disentangle these figures [36]. In some instances, monetized outcomes are not measured creating a gap in our ability to apply economic methods to assist program evaluation [37]. Third, economic analysis of drug prevention is frequently conducted retrospectively [31,38,39]. The problem of data availability is generally more acute when the analysis is planned after the program has already been executed. Hindsight is unlikely to furnish the correct information needed to adequately evaluate a program using acceptable economic principles. Fourth, some researchers resist the idea of monetizing important values such as health and wellness. Critics believe these intrinsic values are absolute and thus should not be reduced to dollars and cents. Furthermore, economic analysis ignores fundamental concerns regarding fairness; a program could provide a positive return on investment even though the majority of its costs are taxpayer burden [40]. Finally, the costs and benefits of prevention programs are often intangible and not easily valued.

Examples of beneficial outcomes from reduced drug use might include improved quality of life and health or reduced mortality and criminality [37,41,42]. These intangible benefits are notoriously difficult to value. For example, the benefits of improved health and decreased mortality must be extrapolated and pieced together from a combination of disease risk factors and assumed morbidity associated with drug use [28,43]. Furthermore, the program’s benefits may not be fully realized for several years or decades after the program, requiring lengthy and potentially costly follow-up analysis [34,39]. In this case, researchers would need to conduct additional analyses to determine the extent and duration of the program’s prophylactic effect on drug use and other outcomes, then calculate a monetized value. For instance, many school-based drug prevention programs have demonstrated durable program effects that extend not only to drug use but include other behaviors including risky driving [44], HIV risk [45], sexual activity [46,47], and delinquency [48].

On the cost side of the equation, a program’s accounting costs frequently require economic adjustment because they exclude important considerations, such as the use of shared resources and volunteers [26,49]. In many cases, school-based drug prevention programs draw heavily on existing school resources (*i.e.*, classrooms), as well as trained teachers and health educators to implement the program [50]. Distinguishing “time on task” for teachers as they prepare lessons and providing efficient estimates of the corresponding dollar values is often murky at best. As a result of these difficulties and others, it is understandable why some researchers may choose not to conduct an economic analysis due to the additional complexity and effort required. However, economic analysis remains a powerful and underutilized tool to estimate the cost-effectiveness or return on investment of school-based drug prevention programs.

### 2.2. Cost-Effectiveness Analysis

Cost-effectiveness analysis (CEA) and cost-benefit analysis (CBA) are the two dominant forms of economic analysis used to study drug prevention programs with CEA by far being the most frequently employed for program evaluation. Cost-effectiveness studies compare the relative costs and outcomes of several courses of action and report these outcomes in noneconomic units; e.g., dollars spent per a given reduction (lowered prevalence rate comparing treated to control youth), delay in drug usage [33], or quality-adjusted life-years [51]. Programs can be compared and contrasted in terms of efficiency in achieving desired outcomes, with those that can achieve desired outcomes at the lowest cost generally being preferred [52]. A program is highly desirable (and thus dominates other alternative choices) when it is both less expensive and more effective. Two concerns should be addressed with CEA studies. First is the “*no single yardstick problem*”, which arises when programs have multiple desired outcomes [28,53].

There are several ways to illustrate this first concern. In one case, a program can have favorable outcomes with marijuana use (lowering reported use by students) whereas another program using the same basic intervention modalities emphasizes decrements in self-reported alcohol use. Each endpoint has different, albeit highly desirable, cost ramifications for society. Programs may also differ with respect to their effectiveness at different stages of drug use. For instance, one program may prevent onset to drug use, whereas another one may influence the progression from early experimental to more problematic levels of use or even deal with cessation. This latter emphasis is consistent with stage models of drug use and may appeal to the end user because of its scientific relevance [54]. Regardless of program differences in outcome or developmental stage emphasis, both sets of programs have been deemed “*evidence-based*” given various professional standards issued by the proper authorities, making the selection between the programs difficult. Notwithstanding, their behavioral specificity, short *versus* long-term benefits, anticipated costs, and political overtones may incline funders to choose one over another.

Furthermore, programs may intend to reduce drug use but indirectly foster other positive outcomes such as reduced criminality or lowered unemployment. In any one of these examples, the techniques used to determine cost-effectiveness do not provide a method to combine the disparate outcomes into a single “*valuation*” metric to compare programs. CEA ratios must then be calculated individually for each outcome measure [26]. The inability of CEA to collapse multiple outcome measures into a single metric is less of a concern when programs have a small number of simple (and often highly related) outputs, but becomes more challenging when faced with multiple and complex outputs, all of which are expressed in non-economic terms. In this situation, it is difficult for policy makers to choose the most optimal program.

A second important consideration with CEA methods is that there are no explicit criteria for program adoption [28]. In other words, while CEA informs policymakers precisely which programs are most efficient relative to some standard (programs can be compared to each other as per the previous example), this approach does not provide sufficient information whether or not the programs provide a worthwhile return on investment to the taxpayer. For example, if a program decreases substance abuse by 10% at a cost of US$1,000 per participant, is the program worthwhile? What if the same program costs US$5,000, US$10,000, or even US$100,000? Cost-effectiveness analysis cannot determine the financial value of these designated program outcomes, but rather must involve the judgment of policymakers and taxpayers to determine whether the cost of programs are worthy of selection and implementation. Researchers need to strongly outline the reasons and justifications for how their programs are socially beneficial and comport with societal expectations of acceptable cost.

### 2.3. Cost-Benefit Analysis

Cost-benefit analysis is the second most common approach to evaluate drug prevention programs in economic terms. This approach resolves both of the limitations encountered with cost-effectiveness analysis by monetizing outcomes that can be expressed in financial terms. The results of cost-benefit analysis are typically expressed as *return on investment* (ROI), which is the ratio of benefits to costs [37,55]. Since the range of program outcomes are reduced to a single monetary denominator, programs can then be compared according to their overall ROI.

The cost-benefit approach provides certain remedies to the limitations of the cost-effectiveness analysis. However, there are still several drawbacks that need mention. In cost-benefit analysis, the data requirements are much steeper and the analysis is more complex when compared with cost-effectiveness. For instance, many prevention program outcomes are intangible and not easily translated into dollar terms. As a result, researchers will need to collect additional data sources and introduce several assumptions in order to properly monetize health outcomes. An example would include establishing the durability of program effects through longitudinal follow-up. Such activity requires extensive data collection and then appropriately monetizing the current *versus* future value of behavior through a discounting process [31]. In addition, the translation to dollars of quality of life improvements and other intangibles may be controversial and highly sensitive to differences in measurement methods.

There is also the potential for bias by researchers who can choose different economic methods to value their program. This presents a major hurdle with differing approaches and cost valuation methods leading to different findings. Furthermore, cost-benefit analysis does not address the issues of fairness; costs and benefits may not be equitably distributed across society [28,56]. Two concerns surface with regard to allocation of resources and their impact on policy decisions. On the one hand, most primary or “universal” prevention programs are offered to entire schools with the goal of boosting student refusal and personal skills to offset vulnerability to drug use. Even with the best of intentions, it is likely that most youth don’t currently use drugs, have no intention of using drugs, and are not vulnerable to drug use based on traditional contextual, individual, and interpersonal factors linked etiologically with drug use [57,58]. Thus incredible expenditure can be earmarked for a population that has no identified pattern of risk with the hope of a “waterfall” effect; covering as much as possible with scarce resources. This may dissuade policy makers from purchasing programs (or voting in favor of funding large-scale research trials to test program efficacy) unless there is a corresponding well-identified public health concern.

In a related vein, many indicated prevention programs target “*high-risk*” or socially marginalized youth who lack access to appropriate community services. Without community support, research funding, or investment (buy-in), these types of programs would not traditionally find a home in these highly specialized environments lacking social capital. Recent efforts have examined the utility of creating sustainable community-university partnerships [59,60], but this requires dutiful monetizing and gauging the economic reality of certain intangibles. Added to these concerns, certain programs impose differential costs and benefits on specific groups compared to others, such as men *versus* women [51,53]. These limitations, in addition to the added time and costs to conduct a full cost-benefit analysis, help to account for the dominant role of cost-effectiveness studies in the literature. However, cost-benefit analysis provides a more complete picture to policymakers and allows benefits of the program to be directly compared with the costs incurred.

## 3. Major Cost Drivers

With these analysis tools in mind, one can then turn to the derivation of economic costs for drug prevention. Numerous methodologies exist in the literature to calculate program costs. Some of these diverse approaches have been discussed by French and colleagues in the Drug Abuse Treatment Cost Analysis Program and also by Gold *et al*. in their discussion of the Guiding Principles for cost analysis in health studies [52,61]. This next section examines some of the most common cost drivers in school-based drug prevention and highlights some of the established practices for calculating costs.

The costs of school-based drug prevention programs may be broken down into two uniquely different types of costs. *Direct costs* are borne by the program itself and could potentially include the cost of paid personnel, participant recruitment and screening, training, supplies, and purchased or rented equipment and facilities. *Indirect costs* include costs that are not directly charged to the program but are instead borne by others. These costs may include volunteer time, donated goods and services, shared facilities, costs borne by participants and their families, and staff time of school teachers and administrators [41]. When direct and indirect costs are combined they represent the full opportunity cost of the prevention program.

### 3.1. Personnel

Personnel costs generally represent one of the largest cost drivers for drug prevention programs. This cost element includes both the salary and fringe benefits of four distinct groups: program staff, school staff, participants, and volunteers. This distinction is made to separate individuals outside the school system who may be responsible for program development and implementation. This collection of personnel usually entails the program developer, research, development, and evaluation team. Programs traditionally report only the cost of program staff. Aos *et al*. have also argued that excluding the substantial costs to other groups is a defect in the evaluation evidence for drug prevention programs [38]. Program developers generally argue that these latter costs should not be included since the schools would have to pay teachers’ salaries regardless of the program, and volunteers and participants’ families provide their time free of charge. This approach ignores the concept of opportunity cost—the teachers could have spent their time teaching another program (or gaining added experience to teach their assigned academic curriculum), volunteers could be contributing towards some other worthy cause, and families may have to reduce working hours or sacrifice their leisure time in order to participate in the program [38]. An emphasis on opportunity costs suggests that resources sacrificed for the program should be valued at market rates, even when they are considered donations or “free” resources [26,27].

In terms of valuation, personnel costs include not only salaries and wages, but also fringe benefits, which approximate 21.5% of the annual salary for public school workers in the United States [62]. If exact salary figures are unavailable, a wage rate should be estimated based upon the age and gender of the individual [52]. For program staff, costs may be extracted from traditional accounting records. However, accounting records are insufficient to estimate the costs from other personnel categories. Teachers may spend a substantial amount of their time involved with training, preparation, and execution of the program [36,63]. Researchers must not only determine the salaries and benefits provided to school personnel, but also the number of hours spent on activities related to the program [64].

Volunteer time may also be a major category of personnel costs and significantly drive costs in school-based drug prevention programs. Chatterji *et al*. estimated the cost of volunteer time using the hourly wage rate of teachers and teacher’s aides [36]. Drummond *et al*. applied a different approach using the local market rate for unskilled labor [26]. Interestingly, Swisher *et al*. found that two-thirds of cost-benefit estimates did not include the value of donated time [32]. The value of volunteerism in school-based interventions may be low relatively to other delivery formats, perhaps justifying their frequent exclusion. Costs to participants and their families may be accounted for if necessary, but these are uncommon when the intervention is delivered exclusively in a classroom setting. This is quite different for school-based programs containing a family component that may involve extensive travel, loss of economic productivity when parents attend sessions, and displacement of resources when parents pay babysitters to care for children not attending the program [65].

### 3.2. Supplies and Course Materials

Prevention programs frequently require participating schools to purchase materials to facilitate the program. To illustrate, Caulkins *et al*. reported the costs of materials for Life Skills Training and Project ALERT on a per class basis, costing US$161 and US$76, respectively (in 1992 dollars) [39]. The cost of course materials can also be annuitized for the length of their expected usefulness over several years of implementation. Additionally, schools may receive donations of goods or services that are consumed during execution of a program. The Drug Abuse Resistance Education (D.A.R.E.) program uses this approach providing free bumper stickers, handouts to students (book covers), and providing the instructing police officer at no charge to the school district [66]. In most cases, the value of a donated resource can be calculated by multiplying the share of that resource used in the program by the fair market value of that resource [27].

### 3.3. Capital Equipment

Capital equipment is frequently purchased at the beginning and used throughout the life of the program. Unlike supplies and course materials, capital equipment is expected to last multiple years and often carries a significant cost. Common examples in prevention programs include office furniture, computer equipment, software for program evaluation (statistical analysis), and facilities (if purchased) [27]. These items require special accounting to spread their costs over the useful life of the equipment, or else the initial costs of the program will appear inflated. Estimation of equipment costs has two distinct parts—the opportunity cost in terms of the funds used to buy the capital, and the depreciation of the equipment. Equipment should be valued at market rates, and the preferred method of accounting for equipment costs is to annuitize the initial capital cost over the useful life of the asset using the Equivalent Annual Cost (EAC) method [27,36]. The EAC method has the advantages of capturing both the depreciation and opportunity cost in a single computation. To use EAC, researchers must estimate the rate of return that could have been generated if the funds had been invested elsewhere, and the depreciation rate for the equipment. The cost of the equipment over the program period is the combined value of the foregone investment income (gains from other use of the money) and depreciation (loss from use of the equipment over time). The usage of rental rates or Internal Revenue Service guidelines for depreciation accounting is an acceptable but less preferred option. Another wrinkle in the analysis of equipment costs occurs because the physical life of the equipment and the useful clinical life may differ. For example, a computer system may be expected to function for five years but considered outdated after two or three. Drummond *et al*. provide a more detailed analysis of the EAC method and address the utility of using conservative estimates to account for equipment costs [26].

### 3.4. Infrastructure

Economic analysis must also include the rent, utilities, and overhead costs that are attributable to the program. Such infrastructure costs are generally difficult to identify and calculate because many of the services and facilities used to implement the program are shared with the school. Program developers may be tempted to exclude school infrastructure costs; however, this ignores the opportunity cost of infrastructure that could be used for alternative programs. There is no one right way to handle these shared costs, leaving wide latitude in determining the best method for cost allocation. Direct, simultaneous, and step-down allocation methods represent three options to assign overhead costs to the program according to a specified parameter [28]. Caulkins *et al*. used the direct allocation method to first estimate the schools’ annual cost of instructional staff and facilities, and then divided the total cost by the number of class sessions that were taught during that year [39]. This method yielded a cost per session estimate, which was then multiplied by the number of sessions required to teach the program. Step-down and simultaneous allocations are more computationally complex by allowing for the interaction between different overhead departments. These latter methods attribute some of the overhead costs to different overhead departments themselves (e.g., janitorial staff, security, school administration). In using any of these different methods, researchers must balance the desire for accuracy with the added costs and difficulty of exercising step-down and simultaneous allocation methods. Cruder allocations methods may be suitable in the analysis if the results are shown to be insensitive to the methodology used, but are not preferred [26]. In the less likely event that the program has to purchase infrastructure, costs should be annuitized over the course of its useful life using the EAC method, in the same manner as capital equipment.

### 3.5. Research & Development (R & D)

The costs to initially develop, test, refine, and retest a drug prevention program may be considerable. Additionally, ongoing program evaluation and program surveillance (e.g., tracking implementation fidelity), if applicable, add to the final total. In some cases, R & D expenditures are “*sunk costs*” that cannot be recovered even if the program is halted before full implementation. In certain cases, economic analyses exclude estimating costs for research (e.g., program evaluation) because it not part of a permanent program. Indeed, Swisher *et al*. found that only one of nine cost-benefit analyses of drug prevention programs included the costs for program development and evaluation [32]. In the one study cited, R & D cost US$925,000 in 1989–1990 and comprised approximately 41.3% of the total cost of the intervention in that year [28]. Because the up-front cost of programs may be considerable, inclusion of R & D costs may place a serious downward bias on a program’s return on investment. However, it is prudent for the researcher to present cost figures both with and without R & D as part of sensitivity analysis and program transparency. If possible, the costs of development and evaluation may be amortized over time and across new populations for the expected length of the program.

## 4. Translating Outcomes to Dollars

### 4.1. Cost Avoidance

Researchers conducting cost-effectiveness analysis may avoid the tricky step of assigning dollar values to program outcomes. The results of CEA studies are presented as a series of ratios of cost per outcome; e.g., dollars spent for each unit reduction in substance usage, or delay in onset of use. In contrast, cost-benefit analyses present a ratio of benefits to costs. CBA does not assume that a reduction in drug use has intrinsic value, but instead derives value indirectly through other related outcomes such as improved health, lower crime, or reduced spending on social (palliative care) services for children of drug-abusing parents. Researchers conducting a cost-benefit analysis must first calculate the value of these related outcomes in order to determine how reductions in usage provide value to the participant and society.

A fundamental concept to CBA studies of school-based drug-prevention programs is *cost avoidance*. Cost avoidance is loosely defined as actions taken to reduce future costs. These actions may potentially incur higher costs in the short-term, although the long-run cost reductions are expected to outweigh these immediate expenses. One example of this consideration may be found in childhood vaccinations. Parents incur immediate costs with the expectation that the vaccination will prevent future, more costly illnesses. School-based prevention programs function under the same guiding principle; programs expend resources on prevention in order to avoid the burden of substance abuse in the future. Cost avoidance is frequently mistaken with *cost savings*, although this latter term denotes tangible and more immediate cost reductions. Cost avoidance is more frequently used when the reductions are less tangible and may not be realized for extended periods of time. For example, drug prevention programs can be administered to participants during adolescence yet are expected to lead to better health outcomes during young adulthood and even beyond.

The majority of calculated benefits in CBA of school-based drug-prevention programs are related to cost avoidance. These programs partly ameliorate the enormous problems induced by substance use, abuse, and dependency [43]. The economic cost of drug abuse in the United State was estimated at $180.9 billion per annum in 2002. These costs have increased steadily at approximately 5.3% per year from 1992 to 2002, slightly outpacing the growth in GDP. A government-funded study estimated the cost of alcohol abuse as US$185 billion per annum in 1998 [67]. A more recent Center for Disease Control and Prevention study estimated the economic costs of excessive alcohol consumption as US$224 billion per annum in 2006 [68]. The latter figure included information from national databases providing guidelines to estimate health care (including conditions directly attributable to alcohol), workplace productivity losses (e.g., work related absenteeism), criminal justice and other related costs (e.g., motor vehicle crashes). There are two primary approaches used to analyze the costs of substance use; the human capital approach, and the willingness-to-pay approach. The remainder of this section outlines each approach, benefit categories traditionally included in economic analyses of drug prevention, and relevant methodologies to capture these benefits.

### 4.2. The Human Capital Approach

The human capital (HC) approach is the most common approach used in CBA of school-based drug-prevention programs, and CBA health and medicine in general [69]. The HC approach looks at the total value of all resources used or lost as a result of actual or anticipated adverse impacts of drug and alcohol dependence or abuse [70]. This approach has been criticized because it assumes a lower value for the young, old, and minorities due to their lower earning potential [28].

The cost-of-illness (COI) methodology is traditionally used to calculate costs for the human capital approach. COI conveys the aggregate burden of substance abuse on society by modeling the costs of treatment, impacts on quality of life, and losses in worker output and productivity. This method requires knowledge about the incidence rates of disease, expected courses of treatment, mortality and morbidity, and the impact of the illness on earnings [64]. Drug and alcohol abuse require the COI methodology to be extended since they lead to additional health problems that must be treated [67]. In other words, the COI methodology has to consider the costs of tobacco use on respiratory problems or kidney disease that may surface in later years and directly result from tobacco or nicotine addiction. CBA practitioners frequently use secondary data and government health care resources to determine these costs due to the immense difficulty and complexity of the task. Although not specifically an economic analysis of school-based drug prevention (the program was family-based but randomization to experimental condition was at the school level), Spoth, Guyll, and Day used prevalence rate information from a large nationally representative study of alcohol disorders to compute the costs experienced by alcohol abusers to society (in terms of health) and the rates of individuals who experienced alcohol abuse/dependence for the representative age group [71]. These numbers were used to gauge the effect of the family-based treatment on youth, in terms of cost avoidance (the plentiful savings from implementing the program and curtailing alcohol use) and also establish the “normal” rate of youth that might become alcohol abusers had they not been treated (in the absence of treatment how many youth would drink and progress to alcohol disorder based on national estimates).

### 4.3. The Willingness-to-Pay (WTP) Approach

The WTP approach represents the sum total of what people are willing to pay for an increase in health or a reduction in illness [68]. Economists adhering to this approach utilize contingent valuation methods (CVM), which are a collection of survey-based economic techniques that allow a researcher to place a value on a good, service, or resource where market prices do not exist. Consider the following premise: It is unlikely that an individual can frequent a store and pay money to reduce the number of drug users. In light of this, CVM represents the primary methods used to determine the value of making this reduction occur. In a CVM study, respondents are presented with a hypothetical narrative that includes the costs, benefits, risks, and other relevant information that allows the reader to visualize the full breadth of the program and its expected health improvements. Respondents are then asked a series of questions to determine how much value they place on the program.

The value that individuals place on the desired health improvements is their willingness-to-pay, the maximum amount they would be willing to personally contribute to support the program. These “bids” can be aggregated and extrapolated to the rest of the affected population to determine the program’s total value. Researchers must assume that respondents’ bids are accurate and represent the true value that they place on the program. Attempts to validate this assumption are plagued with challenges. This technique is generally used to value goods and services that are not traded in the marketplace and ipso facto do not have sufficient market information that could be compared with their hypothetical valuations. To address this challenge, Chang, Lusk, and Norwood conducted an experiment that compared the results of hypothetical CVM surveys with actual shopping behavior for several different grocery items and found that the results closely matched real-world behavior [72]. However, earlier experiments have found that respondents generally overstate their WTP [73], leading to the development of special survey techniques to reduce this upward bias [74,75].

Ignoring the potential lack of similarity between survey results and actual behavior, the WTP approach is difficult to implement and rarely used due to the complexity and survey effort involved [28]. Additionally, the WTP approach presents several issues that may affect the validity of bid amounts. For example, the “embedding effect” is a term denoting when respondents provide similar WTP bids across different surveys, even when economic theory suggests or requires that they be different [76]. This phenomenon may occur when respondents fail to consider their individual budget constraints when answering the survey, or if respondents are reporting what they are willing to contribute towards the good in general (e.g., “health”) and not the specific health improvement described in the survey. Another issue with the WTP approach is the potential for implausible responses. In the environmental literature, respondents frequently provide positive bid amounts to preserve or clean up parks, lakes, and other places that they will never visit. If these surveys were distributed nationwide, the total amount given for a single improvement frequently reaches into the billions of dollars range [73]. For a more complete description of the limitations of this approach, please see Diamond and Hausman or Arrow *et al*. [73,76]. 

### 4.4. Cost Avoidance Benefit Types

Building on the theme of cost avoidance, benefits of prevention come through reductions in several different categories of costs. These “*benefits*” are typically broken down into four categories; healthcare, crime, productivity, and social welfare. In terms of healthcare costs, three additional subcategories can be addressed: prevention efforts, alcohol & drug treatment services, and co-morbid conditions. Expenditures related to substance abuse prevention are substantial and estimated at US$2 billion in 2005 [1]. However, these costs are typically excluded from economic analyses because inclusion of prevention-related costs would artificially inflate the costs of substance abuse. In essence, costs of the solution are being added to the costs of the problem. Direct treatment of substance abuse by community-based and federal facilities amounted to more than US$6.2 billion in 2002.

#### 4.4.1. Healthcare

Researchers may utilize the treatment costs from an average drug user in order to calculate the program’s benefits in terms of avoided treatment costs (e.g., [28,37]). Additionally, drug abuse may complicate treatment of other health or psychological conditions and contribute towards other illnesses such as tuberculosis, HIV/AIDS, hepatitis B & C, and in the case of parental drug abuse create drug-exposed infants. Total healthcare costs of drug and alcohol abuse to society in 2002 were estimated at US$16 billion per annum and rising 4.1% annually [63]. Substance abuse was responsible for two out of five cases treated in psychiatric hospitals, 22% of Medicare expenses, and 23.8% of Medicaid expenses [77,78,79]. Studies have used the COI methodology to determine the partial impact of substance abuse on the prevalence, severity, and treatment complexity of other conditions and have used this data to estimate program benefits in terms of reduced healthcare expenditures [36,80].

#### 4.4.2. Crime

Substance abuse also imposes costs on society through increases in criminal behavior. These costs manifest themselves in criminal justice system expenditures, legal defense, and costs to victims. The Office of National Drug Control Policy reported in 2004 that substance-abuse related criminal justice costs in 2002 totaled US$35.3 billion, legal defense totaled US$647 million, and property damage to victims totaled US$206 million [67]. An earlier report estimating losses to victims by Harwood *et al*. indicated higher costs (US$726 million) by including victim healthcare costs [70]. It is possible that victim costs are greatly undervalued; out of 5.25 million violent victimizations reported nationwide in 2002, 380,000 (7.2%) were attributable to substance abuse [67]. Additionally, driving under the influence is responsible for nearly ten thousand traffic fatalities, several hundred thousand crashes, and more than a million arrests each year in the United States [81,82,83]. These different statistics and the failure to include alcohol- and drug-related injuries and driving related fatalities may seriously underestimate the true losses (both monetary and otherwise) to victims. In addition, long-term follow-up is required to correctly estimate the costs of drug use on criminal offenses that may only surface in later years. Zarkin *et al*. and others provide greater detail on the various techniques for modeling marginal costs of criminal offenses attributable to substance use [36,84,85]. In particular, when long-term follow-up information on drug abuse prevention programs is available, researchers utilize group-level differences in criminality along with marginal costs of criminal offenses to calculate the lifetime cost avoidance potential of their respective programs.

#### 4.4.3. Economic Productivity

The United States economy experiences large losses in gross domestic product due to substance use and abuse. The losses in productivity are due to underemployed or unemployed labor resources, and represent the costs involved in hiring someone to perform the services that cannot be performed due to substance-abuse related sickness, disability, incarceration, or death. Estimates of these costs have steadily increased over time; US$82 billion in 1992 [70], US$98.5 billion in 1998 [43], and US$128.6 billion in 2002 [67]. In more ways than one, drug prevention programs may create economic benefits by keeping workers on the job and also out of jail. Programs that reduce incarceration as a consequence of reduced drug use may calculate the value of participants’ additional earnings and add their economic productivity to the program’s benefits [85].

Substance abuse in adolescents has also been linked to poor school performance, early exit from school, and lower levels of college attendance and completion [86]. Several school-based interventions have collected data on high school and college graduation rates to estimate the impact of drug prevention programs on participants’ future earnings [37,87,88]. These studies utilize census data in order to determine the lifetime earnings impact from completing a high school diploma, attending some college but no degree, or obtaining a college degree, controlling for demographic factors. This information is then combined with the program’s outcome data to determine if the program favorably influenced the educational attainment of participants. If so, the program may create an additional benefit in terms of increased future earning potential for participants that were deterred from substance abuse and will now go on achieve higher educational attainment. The value of these additional earnings is calculated to determine the program’s benefit.

#### 4.4.4. Social Welfare Costs

Substance abuse has been tied to increased social welfare costs in the United States. Drug and alcohol abuse leads to higher expenditures in programs such as Medicare, Medicaid, Social Security Supplemental Security Income & Disability Insurance, unemployment insurance, Aid to Families with Dependent Children, and procuring food stamps. Tabulation of substance abuse-related costs is difficult due to limited data on drug usage. Non-health social welfare expenditures were estimated at US$17.3 billion in 1994 [28]. This figure has been criticized because it does not prove that beneficiaries received benefits because of their drug use. More conservative estimates are an order of magnitude less, e.g., US$1.2 billion in 1992 and US$235 million in 2002 [67,70]. Social welfare costs are frequently included in cost-benefit analyses of drug prevention [37,88]. However, Gold *et al*. have recommended in their *Guiding Principles* that social welfare costs be excluded from analysis [52]. While administrative costs may matter, these transfer payments are mere redistributions of wealth. Although they may matter to government decision-makers and some taxpayers, they do not matter from a societal perspective (*i.e.*, the taxpayer has met their burden without recourse to hold back money intended for the Treasury).

## 5. Special Considerations

### 5.1. Analytic Perspective

Clearly stating a perspective for economic analysis in terms of space and time helps to ensure that the costs and benefits considered are relevant to the study sponsor. Costs may differ depending upon the perspective selected for analysis [51]. For example, the agency providing the program may only be concerned about the direct costs of a program. The local civic government may be concerned less with program expenditures, and also with indirect costs borne by the school delivering the program and other agencies that might be involved or affected by the program (e.g., a prevention program reduces referrals for child services). The United States Public Health Service and others have recommended that researchers take the societal perspective and consider all costs in their analysis regardless of which individuals incur these expenses [26,52]. Furthermore, because the societal perspective reflects the broadest stance, the results of the economic analysis may be broken down to lower levels as needed (e.g., national, state, or individual). The societal perspective has been recommended due to several reasons. Using this approach, researchers will be able to identify potential ethical and fairness concerns in the program, such as how costs and benefits are distributed amongst different groups in society. Additionally, there is a practical need to select a single, common perspective in order to compare results across different studies. Other reasons given included the needs of the analysis’ end-users, economic theory, decision theory, and others [52].

Program developers following the societal approach have the incentive to include the widest range of possible benefits in their economic analyses, because it increases the estimated return on investment and makes their programs appear more attractive. Alternatively, an overly thorough researcher may attempt to include the widest range of potential costs. However, the inclusion of more costs benefits may add extra time, cost, difficulty, complexity to the analysis without significantly affecting the results. Enthusiastic researchers must temper their desire to cast as wide of a net as possible and determine if the addition of extra benefits will have a meaningful impact on the results. If certain costs/benefits are especially difficult to determine and aren’t expected to change the results, researchers may rightfully consider their exclusion from the analysis.

### 5.2. Temporal Issues

The costs of a substance abuse prevention program are often immediate yet the benefits frequently accrue later (often much later) in time. However, research shows that it is fairly uncommon for programs to collect the type of long-term follow-up data that can effectively inform economic analysis. In a meta-analysis of prevention programs targeting children and adolescents, fewer than 7% of studies measured outcomes up to one year [89]. The lack of longitudinal post-test data means that long-term durability of program cannot be assessed and should be varied to represent uncertainty. For example, Caulkins *et al*. conducted a sensitivity analysis that tested the differences in program benefits if reductions in substance abuse lasted through high school, mid-life, or death [39]. More recent studies include follow-up extended through young adulthood when crime is more prevalent and costs for productivity losses are of greater economic importance.

Furthermore, costs and benefits that occur in the future must be discounted to a base year using an appropriate discount rate. For costs, the discount rate represents the rate of return that could have been achieved if program funds had been invested in the private sector. For benefits, this discount rate represents the social rate of time preference; goods and services received at an earlier date are valued more highly than those received at a later date [26]. The selection of a discount rate is a very important decision for economic analysis of prevention studies. High rates create conservative estimates by placing higher weights on program costs that occur more immediately and lower weights on benefits that accrue years or decades in the future. On the other hand, lower weights place a relatively higher value on future benefits and make prevention programs appear more attractive. Rate selection may dramatically affect the analysis results, especially over long periods of time. For example, if a program lead to cost avoidance of US$1,000,000 at a time 100 years in the future, a 10% discount rate would reduce the present value of that amount to US$72 while a 3% discount rate would yield US$52,033 [90]. This brings into question the most proper methodology for determining the appropriate discount rate.

For one thing, economists differentiate between the real and nominal discount rate. The nominal discount rate incorporates not only the society’s time preference but also inflation, while the real discount rate excludes inflation. A careful inspection of the existing literature reveals application of widely different rates. Caulkins *et al*., for instance, suggested using a 4% real discount rate because it is “*typical*” or commonly used [39]. Others recommend using the interest rate on government bonds, because that is the rate at which individuals invest and provide funds to the government [52,69]. Most current cost-benefit analyses use real discount rates between 2% to 4% [31,37,77]. Program developers may be tempted to choose a lower discount rate with the expectation that it will improve the economic attractiveness of their programs. However, it is important to temper enthusiasm. The low discount rates used in the existing literature may underrepresent the social rate of time preference and the opportunity cost of program expenditures.

In many respects, using the interest rates on government bonds is justified because this approach matches the government’s borrowing rate; however, these funds are drawn from the private sector through taxation. The government rate is lower than the private rate of borrowing and thus does not fully capture the opportunity cost of those funds. The average interest rate for the private sector may also be too low because it includes industries that receive supranormal returns, monopolies, *etc*. The marginal cost of capital would provide the most conservative discount rate and may be a truer approximation of a program’s opportunity cost of prevention. The marginal rate represents the cost of the last dollar of capital raised, and represents the market rate that a private firm would face if it sought additional funding.

However, economic efficiency is not the sole criterion for determining the appropriate discount rate. The selection of a discount rate may have profound effects on intergenerational equity [90,91,92]. This is especially relevant to drug prevention since the benefits of prevention may not accrue until decades after the intervention has been completed [90,92]. This presents the researcher with a dilemma; what discount rate balances competing concerns about efficiency and equity? This problem has been referred to as the “conservationist’s dilemma” since low discount rates increase the value of natural resources such as forests, which are slow-growing. However, the same low discount rates also increase the attractiveness of investments in the expensive infrastructure required to extract these resources [92]. In practice, there are several alternative courses of action to address this dilemma. The first is to ignore it; the discussion of intergenerational equity is notably absent from existing economic analyses of school-based drug prevention. In other applications, researchers have employed a variety of methods such as selecting an artificially low discount rate [93], selecting the marginal product of capital (the increased output that results from a one unit increase in capital equipment) [94], and the use of differing medium-term and long-term discount rates [95]. Each approach is not without its weaknesses, and a consensus regarding the appropriate adjustment to the discount rate to account for intergenerational equity has been elusive. From a practical perspective, since these different rates may provide dramatically different results it is important for the researcher to conduct a sensitivity analysis using several alternatives.

### 5.3. Effect Multipliers and Scaling Factors

Some studies have used multipliers to increase the calculated benefits of programs. For example, Caulkins *et al*. included multipliers that account for more focused and effective drug enforcement, reduced peer pressure, and reductions in other substances that are theorized to occur due to the program’s effect on use of a particular substance [39]. The enforcement/market multiplier was estimated under the assumption that a reduction in the number of drug users would allow the police to target their enforcement efforts on a smaller drug market, increasing their overall effectiveness. A peer pressure multiplier was included because drug use frequently occurs as a group phenomenon, and thus a reduction in drug users may be expected to alter the normative environment and lead to further reductions (*i.e.*, drug use is no longer perceived as socially acceptable). Caulkins *et al*. also theorized that a reduction in usage of one drug may correspond with reductions in other drugs that were not directly addressed by a particular drug prevention program [39]. This is not uncommon with school-based drug prevention programs that focus on gateway drugs but then show favorable program effects on other illicit drugs in late adolescence or early young adulthood [96].

Alternatively, other studies have included multipliers to decrease program benefits. For instance, scale-up multipliers may represent the expected degradation of effectiveness in a school-based prevention program as it increases in size [31,37,38,39,97]. Furthermore, some studies have included a multiplier to adjust for the stringency of the research and its ability to assess causal effects, with randomized clinical trials representing the gold standard [37,98]. In practice, these multipliers are sometimes arbitrarily decided and dominate the analysis results. For example, the multipliers in Caulkins *et al*. accounted for more than two-thirds of the total calculated value of reductions and delays in substance use [39]. Alternatively, Miller and Hendrie chose to exclude several of these multipliers because of their extremely high uncertainly and potentially spurious nature [31]. Researchers should be hesitant to include a multiplier unless there is strong theoretical and empirical evidence to apply this method.

### 5.4. Importance of Sensitivity Analysis

It should be clear from this brief review that economic analyses of drug prevention include a great deal of uncertainty. Estimates of the economic implications of drug abuse yield precise values; however, these should be viewed as order-of-magnitude approximations. Data is usually gleaned from secondary sources and collected without economic analysis in mind [67]. These secondary sources may lack the detail, granularity, and accuracy that are required to properly conduct an economic analysis. Economic analyses of school-based drug prevention programs are especially uncertain because interventions are often given to preteens while drug usage may not start until some later point in adolescence long after the intervention has concluded. Estimates of these distal outcomes are based upon statistical projections and could be significantly altered by future events. It is of critical importance for researchers to capture this uncertainty in their models by conducting sensitivity analysis, especially on important facets such as discount rate and incidence rates for health condition [52,69]. Results may then be presented as a range of possible values for the program’s return on investment. As an example, Caulkins *et al*. used Monte Carlo simulation to model the uncertainty of their estimates [39]. This method is standard fare in risk modeling and provides information about the distribution of possible costs and benefits and the probabilities that they will occur. Simple implementations of Monte Carlo may be completed in standard versions of Microsoft Excel. Caulkins *et al*. developed low, medium, and high estimates for individual cost drivers, and then created a computer simulation to randomly select one of the three estimates for each. The model simulation was run 16,000 times (with randomly drawn samples), providing the likelihood of particular cost scenarios instead of a single point estimate for program costs [39]. An alternative and simplified technique may be found in Crowley *et al*. and Chatterji *et al.,* both of which employed *extreme-scenario analyses* to describe the best and worst case cost estimates [36,49]. The first step in this method is to determine the minimum and maximum plausible range for each cost element. In doing so, the researchers create an upper and lower bound for their estimates.

While the examples provided in this section limit their analysis to program costs, extreme-scenario analysis and Monte Carlo simulation could be extended to the benefits and the choice of an appropriate discount rate. In practice, researchers could potentially conduct extreme-scenario analyses first to determine if uncertainty has a significant impact on their results, and if so move on to Monte Carlo or alternative methods to account for the uncertainty inherent in their cost and benefit estimates. These methods provide results akin to confidence intervals for estimates of cost-effectiveness or return on investment. Decision-makers and other end-users may use the additional information provided by the confidence interval on results to make their resource allocation decisions.

## 6. Discussion and Conclusions

This article shows that despite their different orientations economic analysis and standard program evaluation of school-based drug prevention programs share numerous common threads. The experimental comparison between treated and “treatment as usual” or control groups, the collection of relevant data fueling program evaluation, tracking youth or cost outlays over time, and the need to precisely demonstrate some level of program “effectiveness” are all hallmark characteristics of both disciplines. Where the two evaluation arms differ is that program efficacy evaluation emphasizes accountability in terms of behavioral program objectives whereas economic analysis emphasizes accountability and efficiency in terms of resource utilization, both monetary and otherwise. For constituents faced with allocating scarce resources both approaches provide much needed information.

Program evaluations of school-based drug prevention have made significant inroads with regard to traditional manipulation checks reinforcing “*treatment construct validity*” [7]; in other words, determining whether the program actually influences the active ingredients that lead to behavior change. The latter emphasis has traditionally relied on health behavior change theories and monitored individual-level responses to select intervention strategies [99]. With the recent societal emphasis on accountability, programs can now also address whether there are monetary and societal benefits to implementing effective programs. In many ways, both economic analysis and program evaluation are “*forward looking*” approaches that deal with anticipated beneficial outcomes. In the former case the emphasis rests with investment opportunity costs (what the money spent on prevention would have yielded if spent in other ways). In the latter case the emphasis rests with anticipated program effects on behaviors that portend tremendous societal savings (*i.e.*, the benefits of drug prevention in terms of reduced crime or lowered health care costs).

To date, economic analyses of school-based drug prevention programs provide clear evidence these programs benefit society by avoiding future costs (in terms of tangible societal costs) and have a high return on investment. Of the handful of cost benefit analyses, most show sizable returns from relatively modest dollars spent. In the case of five efficacious programs delivered entirely in a school setting—Life Skills Training, Project Northland, Project STAR, the Good Behavior Game, and the Project towards No Tobacco Use—the return on investment ranged from US$5.29 to US$55.84 per every dollar spent. Alternatively, the estimated annual costs of the D.A.R.E program range from US$99 to US$270 per student [37,100]; however, the program has not been shown to achieve its desired outcomes [63,101]. A lack of efficacy typically precludes further analysis, since ineffective programs should not be adopted no matter the cost. However, the wide implementation of D.A.R.E. has led to further research on the opportunity cost suffered by communities that adopted the program. Some of these school-based prevention programs are substantially more expensive than others, and these potentially have the most to gain from economic analysis. A high return on investment for a prevention program may be used to overcome the “sticker shock” that policymakers suffer when faced with a more intensive program that may be very efficacious yet entails a high cost to administer.

There are other important parallels to the different approaches to program evaluation. Both disciplines test models with alternative albeit, plausible representations using adjustments that may influence (or confound) outcomes. Economists use sensitivity analyses and simulation procedures to objectively determine the robustness of their methods, whereas program evaluators test covariate adjusted models to avoid mis-specified models, confounding or suppression effects [102]. Cross-validation is also required in both disciplines to assert the internal validity of methods and external validity of findings. Both disciplines assume the end product of their analysis reflects “*rates*” in economic terms reflecting monetized costs for each participant and for behavioral analysis the concomitant reduction in drug use (prevalence rate differences between treated and control students). Furthermore, the economist is interested in ratios that reflect benefits to costs or cost per unit of outcome (e.g., one less alcohol or drug abuser). As a corollary, the behavioral component of program evaluation seeks to establish rates of program success based on lowered incidence of new users following exposure to the intervention and reduced use among those already using drugs at the beginning of the program.

Even with these noted similarities, widespread variation in techniques and evaluation design exists not only between economists and program evaluators, but also within each discipline. This may hinder progress because each field fails to inform the other. Researchers are required to make numerous design decisions when conducting economic analysis, and these decisions may significantly impact their findings. This has led to disparate groups within and between each discipline extolling the virtues of their respected methods without achieving an acceptable consensus. This dispersion of methods is problematic both in terms of the validity of the estimates produced, but also in comparing the results of one economic analysis to another. Gold *et al*. and French *et al*. have been effective in creating guidelines for economic analysis in healthcare and drug treatment [52,61]. However, these seminal works require update and revision not only to account for advances and new ways of thinking, but also to tailor these guidelines to prevention instead of treatment. In large-scale epidemiological studies estimating costs of excessive alcohol use, for instance, reasonable economic costs have used the Guidelines for Cost of Illness Studies in the Public Health Service [103] as a framework. This document draws exclusively from national databases that are accessible, reliable, and readily updated, yielding a “living” source of cost information. Notwithstanding, the success and validity of economic analysis could be greatly improved through the promulgation of even more current guidelines to standardize the process of estimating costs and benefits. These guidelines should comport with the stated criteria produced by program evaluators in their efforts to delineate the composition of evidence-based programs. It is interesting that experts in drug prevention science have called for the use of economic cost principles in determining the “relative impact and benefits” of programs [5]. There are now codified and published “Standards of Evidence” that guide selection and determination of evidence-based drug prevention programs. These standards have experienced a growing presence in the field. There is no better time for these standards to gain traction outside of prevention science and incorporate the rigor and wisdom of economic analyses.

The success of both disciplines also rests with their ability to disentangle information (cost allocation *vs*. behavioral change) in an effort to obtain a more refined view of process. One process involves actuarial applications as youth grow into young adults while the other monitors psychological maturational phenomenon. While the breakdown of how each discipline computes their net effect differs tremendously, the intended goals remain consistent and there is much to be gained from an interweaving of the two approaches. Economic analysis is entirely dependent on information gleaned from traditional program evaluation in order to estimate a program’s effects and benefits [5]. Traditional program evaluation is enhanced with the ability to more effectively compare results across widely varying and disparate programs, as well as information that may be used to justify program adoption and continuation in the face of zero-sum budgetary decisions. More and more, program evaluators are culling results from large multi-site, multi-trial datasets and using this information to establish benchmarks for program efficacy. Both meta-analysis [104,105] Tobler and Stratton, Tobler *et al*.,) and the more recent integrated data analysis [106] (Curran and Hussong) provide the necessary tools to synthesize across disparate findings with the goal of ascertaining what works, for whom, and under what conditions. In this manner, the term “*effective*” takes on a broader mandate when economic analysis is blended with traditional program evaluation, in a way that may redefine what is traditionally considered evaluation science [107].
